# Measuring Impact and Sustainability of Evidence-Based Dementia Care Treatment in the Community

**DOI:** 10.1093/geroni/igaa057.2765

**Published:** 2020-12-16

**Authors:** Jill Cigliana, Kari Burch, Nancy Mueller, Smriti Bajracharya

**Affiliations:** 1 Memory Care Home Solutions, St. Louis, Missouri, United States; 2 Washington University in St. Louis Brown School, St. Louis, Missouri, United States

## Abstract

Through a collaborative agreement with the Administration for Community Living, Memory Care Home Solutions translated the evidence-based program (EBP) Care of Persons with Dementia in their Environments (COPE) into community practice as a covered benefit through Medicare and commercial health insurance for people living with dementia. While many studies have demonstrated the efficacy of family-centered behavioral interventions for this population, few of these interventions have been translated from academic studies into successful community practice. This session will describe the process of translating an EBP into community practice utilizing social work and occupational therapy interventionists with a diverse population of people living in both urban and rural areas of Missouri and Illinois, including modifications required and lessons learned in rural service delivery. Attendees will gain an understanding of the translation process, differential treatment outcomes for people living with dementia and care partners and Medicare billing mechanisms for sustainability.

